# The Effects of Chlorpromazine on Reproductive System and Function in Female Rats

**DOI:** 10.22074/ijfs.2015.4553

**Published:** 2015-10-31

**Authors:** Zahra Zamani, Samad Zare, Rajabali Sadrkhanlou, Abbas Ahmadi, Elham Movahed

**Affiliations:** 1 Department of Biology, Faculty of Science , Urmia University, Urmia, Iran; 2 Laboratory of Embryology, Department of Basic Science, Faculty of Veterinary Medicine, Urmia University, Urmia, Iran

**Keywords:** Chlorpromazine, Hyperprolactinemia, Ovary, Atresia, Rat

## Abstract

**Background:**

Chlorpromazine (CPZ), an antipsychotic drug, is associated with increased risk of sexual dysfunction through increasing prolactin levels. The current study evaluates the effect of CPZ-induced hyperprolactinemia on ovarian follicular growth, gonadotropins, and alteration of ovarian source hormones.

**Materials and Methods:**

In this experimental study, animals were divided into four groups, control and CPZ (n=8 per group). In the treated groups, CPZ was administered by gavage at doses of 3, 10 and 30 mg/kg per day for 28 days. On day 29 the animals were killed after which histopathological and histomorphometric analyses of the ovaries were performed. We evaluated the levels of prolactin serum, luteinizing hormone (LH), follicle-stimulating hormone (FSH), estradiol (E_2_) and progesterone.

**Results:**

The ovaries of the test groups showed numerous atretic follicles of various sizes. CPZ caused a significant difference between the test groups and the control group (P<0.05) on the amount of atresia and the size of the normal corpora lutea (CL). The increased dysfunction of the ovaries from the different groups depended on the amount of CPZ administered. The serum concentrations of prolactin and progesterone significantly increased (P<0.05), while the serum concentrations of estradiol, LH and FSH notably decreased (P<0.05), depending on the CPZ dose. CPZ-induced animals had unsuccessful mating and decreased pregnancy rate.

**Conclusion:**

The present findings suggest that CPZ-induced disturbances not only depend on prolactin level but the increased prolactin level is largely dose-dependent.

## Introduction

Chlorpromazine (CPZ), an antipsychotic drug, has been widely used to treat schizophrenia and other psychotic disorders. CPZ is also used to control nausea, vomiting, long-term hiccups and as treatment for acute intermittent porphyria (1, 2). The antipsychotic effect of CPZ and other types of antipsychotic drugs is on the dopaminergic neurons of the mesolimbic system which is linked with psychotic symptoms (3).

Antipsychotic medications effectively diminish the intensity of psychotic hallucinations and allow most institutionalized patients with schizophrenia to be discharged into community treatment. The use of antipsychotic medications implicates a difficult trade-off between the benefit of alleviating psychotic symptoms and the risk of troubling, sometimes life-shortening adverse effects (4). All antipsychotic medications are associated with increased risk of sexual dysfunction, postural hypotension, cardiac arrhythmia, and sudden cardiac death (5-9). In order to successfully treat patients with schizophrenia, the adverse effect profiles of these medications should be taken into consideration. Physicians should be careful about the occurrence of adverse effects and be willing to adjust or change medications as needed or work with other psychiatrists to familiarize themselves with others’ experiences to enable better and less dangerous treatments (4).

Until recently, increased prolactin rate (hyperprolactinemia) as a common side effect of antipsychotic treatments, has received little attention (10). Antipsychotic drugs block dopamine D2 receptors on lactotroph cells in the anterior pituitary gland and thus remove the inhibitory influence on prolactin secretion (11). Researchers have shown the adverse effect on fertility, sexual function, and bone mineral density of hyperprolactinemia (8, 12, 13).

Prolactin can suppress gonadotropin-releasing hormone (GnRH) secretion from the hypothalamus and directly affect the physiological actions of the pituitary. Prolactin causes gonadotropins [luteinizing hormone (LH) and follicle-stimulating hormone (FSH)] to adversely affect the gonads (12). On the other hand, physiologic function of follicular growth and granulosa cells mainly depend on serum levels of FSH and LH. Therefore the dysregulation of hormones in which their source is ovarian, will lead to important problems in fertilizing potential (13, 14). This disorder in the function of gonadotropins is related to the pituitary gland and its feedback mechanisms.

Antipsychotic treatment is often initiated when patients are in their late teens or twenties. This treatment continues for years or decades (11). Although conventional antipsychotic drugs elevate prolactin rates (above the normal limit for both men and women), no reliable study that shows the relationship between these medications’ doses and the effects of antipsychotic drug-induced follicular atresia is available. Thus, the present study evaluates the dose-dependent effects of CPZ on serum prolactin, sex hormone concentrations and ovarian tissue of adult female rats.

## Materials and Methods

### Animals

We conducted an experimental study on 32 female Wistar rats that were 70 days old and weighed 160 ± 5 g. Rats were obtained from the Animal House at the Faculty of Science, Urmia University, Iran and were allowed to acclimatize in an environmentally controlled room with a temperature of 22 ± 2˚C and a 12 hour light/12 hour dark schedule. Standard pellet food and tap water were available ad libitum. In this study all experiments conducted on the animals were in agreement with the Urmia University guidelines of the Ethical Committee for research on laboratory animals. Animals were allowed to acclimatize for one week before the experiments.

### Drugs

CPZ (Sigma-Aldrich Co., Germany) was used at three dose levels -3, 10 and 30 mg/kg based on previous study (14). The drug was dissolved in 0.5% methylcellulose solution (15) and administered to female rats by oral gavage.

### Drug treatment

After a one-week acclimation, we assigned the animals to four groups (n=8 per group), as control and test groups. The control group rats received 5 ml/kg of 0.5% methylcellulose solution once daily for 28 consecutive days. The test subgroups received 3, 10 or 30 mg/kg/day CPZ for 28 consecutive days. In each group, we randomly chose 4 animals for potential fertility assessment. The remaining 4 animals were used for histological examinations.

One day after the last drug treatment, 4 animals from each group were killed by CO_2_ inhalation and blood samples were collected from the jugular veins. Subsequently, the serum was harvested and frozen. The ovaries were removed surgically.

Prolactin is a stress hormone. Hence, in order to obtain unstressed levels of prolactin, we choose rapid decapitation as the method of sacrifice due to its decreased stress for the rodent. In addition, the animals were not in the presence of one another at the time of sacrifice (to smell the blood).

### Potential fertility assessment

One week before the end of the treatment period, we randomly selected 4 females from each group to be placed in individual cages with one samestrain sexually active male. We considered the day which sperm was detected in smears to be day 0 of pregnancy; after 21-23 days (pregnancy period in rats) the neonates were counted.

### Histomorphologic analyses

On day 29, the ovaries were removed and fixed in formaldehyde acetic solution (IFAA, Merck, Germany) for 4 weeks. Ultimately, they were dissected free from ovarian tissues. Samples were processed through paraffin embedding and serially cut with a rotary microtome (Microm GmbH, Germany), then stained with hematoxylin and eosin (Merck, Germany).

We characterized the follicles in the ovarian sections according to size: under 100, 101-200, 201-300, 301-400, 401-500 and larger than 500 μm. Follicular morphology was examined by microscope under a ×40 objective lens (Olympus, Germany) magnification. Follicles with a complete layer of flattened granulosa cells, a normal nucleus, and oocytes with cytoplasm were considered normal follicles. Abnormal follicles were classified as follows: pyknotic nucleus, cytoplasmic damage, and combination of damaged nucleus and cytoplasm. Follicular number was estimated by counting follicles in all slides (16). The corpora lutea (CL) number per ovary was counted.

### Hormonal assay

Blood sera were separated by centrifugation at 3000 g for 5 minutes, then subjected to assessments of serum levels of LH, FSH, progesterone, estradiol (E_2_) and prolactin. Animals were killed and blood samples obtained in the morning hours.

### Radioimmunoassays of prolactin, LH and FSH in sera

We added 100 μl of sera to tubes which contained 100 μl of hormones labeled with rabbit antisera in 0.01 M phosphate buffer (pH=7.6). Antirat prolactin (Cisbio Bioassays, France), LH and FSH were diluted to 1:5000, 1:10000 and 1:2500, respectively. Goat anti-rabbit IgG at a dilution of 1:10 (200 μl) was added to the mixture after which the mixture was allowed to remain for 18 hours at 40˚C, then centrifuged at 2000×g for 30 minutes. Radioactivity levels in the resultant pellets were measured by a gamma counter.

### Radioimmunoassays of serum estradiol and progesterone

Concentrations of serum estradiol were measured by CIS kits (Cisbio Bioassays, France) according to the manufacturer’s instructions. Serum (300 μl) was extracted with 3 ml ethyl ether. The layer of ether was evaporated under N_2_ gas and the extract resuspended in 300 μl of 0.04 M phosphate buffer. After the addition of 100 μ1 17/3-estradiol (14000 cpm). Goat anti-rabbit r-globulin (1 ml) was added and the mixture was allowed to incubate for 15 minutes at room temperature. After centrifugation, the radioactivity in the pellet was counted. In order to evaluate serum levels of progesterone, we mixed serum (0.1 ml), l ml ethyl ether and 50 μ1 propylene glycol. After evaporating the ether under N_2_ gas, 0.5 ml phosphate buffer and 0.1 ml (20000 cpm) of iodoprogesterone were added to the tube and the mixture was incubated with 0.1 ml anti-serum raised in rabbits for 18 hours at room temperature. Then, 0.1 ml bovine serum gamma globulin and polyethylene glycol were added to the mixture. The mixture was centrifuged for 10 minutes at 2000×g. The radioactivity was measured in the pellet (17).

### Statistical analysis

Data are presented as mean ± SD. Experimental data were analyzed by analysis of variance and Duncan’s multiple range test (SPSS version 16, Chicago, IL, USA).

## Results

### Fertilizing index and neonates

We analyzed the fertilizing index in the control and test groups. In CPZ-administered groups, the two high doses had a negative fertilizing index; these groups produced no neonates. In contrast, the control animals and the 3 mg/kg/day showed positive fertilizing indexes with 35 (control) and 21 (low dose) neonates (Table 1).

### Hormone concentrations

Biochemical analyses showed that the serum levels of prolactin significantly (P<0.05) increased in CPZ-administered animals. This increase was dose-dependent. In contrast, control animals had constant prolactin levels. The serum levels of LH and FSH between the CPZ and control groups showed that the serum levels of LH and FSH remarkably (P<0.05) decreased in animals that received CPZ. This reduction in LH and FSH levels was CPZ dose-dependent. The serum levels of estradiol significantly (P<0.05) decreased, while the progesterone level remarkably (P<0.05) increased in animals that received CPZ, which was dose-dependent. The data for hormonal analyses are presented in table 2.

### Ovarian follicular growth, atresia and corpora lutea

**Fig.1:**
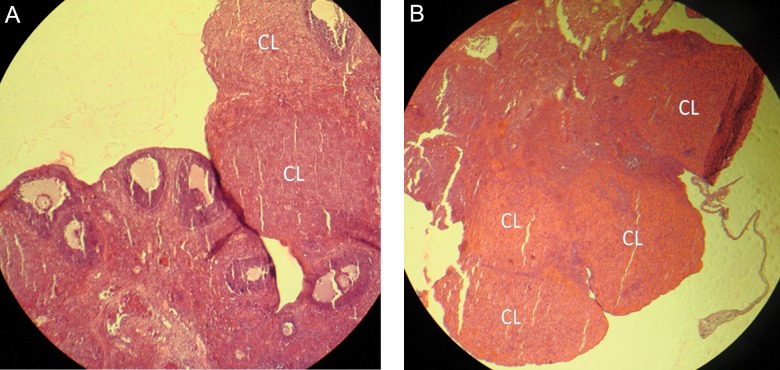
Cross-section from an ovary. A. Control group ovary presents with different size follicles and corpora lutea (CL) and B. 30 mg/kg dose chlorpromazine (CPZ) group show large, active CL without follicular growth. Hematoxylin-eosin staining, (×400 magnification).

Histological analyses in this study showed that in CPZ-administered groups, the total number of normal follicles significantly (P<0.05) decreased compared to control animals. Ovaries from the control group contained follicles in various developmental stages including primordial, primary, secondary, tertiary and graafian follicles with different sizes that ranged from <100 μm to >500 μm. There were no large antral follicles (>500 μm) in the two groups that received high doses of CPZ. Treatment with CPZ resulted in a significant (P<0.05) decline in follicular size in the CPZ groups compared to the control group. In the CPZ groups, there were more total numbers of atretic follicles compared to the control group. This finding was dependent on the dose of CPZ (Fig .1). Comparing the rate of normal follicles between the control and CPZ groups showed a significant (P<0.05) decrease in the CPZ groups. The highest rate of number of normal follicles between the test groups was observed in the low dose group. We observed that animals which received the two higher doses of CPZ exhibited significantly higher CL sizes compared to the control group (Tables 2-5).

**Table 1:** Fertilizing index (pregnant rats) and numbers of neonates in control and CPZ-treated groups


Parameters	Control	3 mg/kg	10 mg/kg	30 mg/kg

Number of animals examined	4	4	4	4
Mated animals (n)	4	4	0	0
Fertility index (%)^1^	100	75	0	0
Neonates (n)	35	21	0	0


^1;^ Fertility index (%)=(number of pregnant animals/number of animals that copulated)×100 and CPZ; Chlorpromazine.

**Table 2:** Mean serum levels of prolactin, LH, FSH, progesterone and estradiol (E_2_) in study groups


Hormones	Control	3 mg/kg	10 mg/kg	30 mg/kg

Prolactin (ng/ml)	55.75 ±3.06	109.25±13.37	223.75±26.35^a,b^	249.50±25.82^a,b,c^
LH(ng/ml)	0.56±0.05	0.58±0.06	0.30±0.02^a,b^	0.26±0.02^a,b^
FSH(ng/ml)	3.17±0.48	1.97±0.44	1.35±0.27^a,b^	1.13±0.06^a,b^
E_2_ (pg/ml)	41.50±2.62	29.00±1.47	29.50±2.59^a,b^	24.00±0.40^a,b^
Progesterone (ng/ml)	18.12±2.55	22.75±3.11	32.07±3.75^a,b^	33.82±3.71^a,b ^


^a, b, c^; Indicate significant differences (P<0.05) between data of chlorpromazine (CPZ) groups with control, 3 mg/kg and 10 mg/kg groups, respectively. All data are presented as mean ± SD. LH; Luteinizing hormone, FSH; Follicle-stimulating hormone and E_2_; Estradiol.

**Table 3:** Mean numbers of normal and atretic follicles on ovaries of study groups


Parameters (n)	Control	3 mg/kg	10 mg/kg	30 mg/kg

Primordial follicles	287.50±11.90	250.75±13.47	137.50±4.19^a,b^	154.75±9.46^a,b,c^
Primary follicles	4.75±0.48	3.75±0.48	5.00±0.57	6.50±0.64
Secondary follicles	7.00±0.70	4.50±0.28	4.50±0.28^a,b^	3.75±0.62^ ,a,b^
Tertiary follicles	6.50±0.25	7.50±0.28	5.75±0.47	5.75±0.75
Graafian follicles	8.25±0.75	7.00±0.40	5.50±0.28^a,b^	3.75±0.85^a,b,c^
Atretic follicles	1.25±0.25	3.00±0.41	11.50±0.29^a,b^	12.50±0.64^a,b,c^
Preantral atretic follicles	0.25±0.25	1.25±0.25	5.75±0.48^a,b^	7.00±0.41^a,b,c^
Antral atretic follicles	1.00±0.00	1.75±0.29	5.50±0.57^a,b^	5.50±0.70^a,b,c^
Corpora lutea	10.50±0.28	10.75±0.62	11.50±0.57^a,b^	10.50±0.64^a,b^


^a, b, c^; Indicate significant differences (P<0.05) between data of chlorpromazine (CPZ) groups with control, 3 mg/kg and 10 mg/kg groups, respectively. All data are mean ± SD.

**Table 4:** Sizes of follicles on ovaries of different groups


Follicles (µm)	Control	3 mg/kg	10 mg/kg	30 mg/kg

<100	294.00±10.97	255.25±13.82	148.00±4.70^ a,b^	165.75±9.93^ a,b,c^
100-200	5.50±0.86	6.00±0.70	12.50±1.29	9.00±2.27
201-300	6.50±0.22	7.50±0.64	9.75±0.75	6.25±0.63
301-400	6.75±0.47	7.00±0.70	8.50±0.86	6.00±0.12
401-500	2.50±0.44	3.00±0.58	2.25±0.25	2.50±0.86
500<	1.25±0.62	0.75±0.25	0.00±0.00	0.00±0.00


^a, b, c^; Indicate significant differences (P<0.05) between data of chlorpromazine (CPZ) groups with control, 3 mg/kg and 10 mg/kg groups, respectively. All data are mean ± SD.

**Table 5:** Sizes of corpora lutea (CL) in chlorpromazine (CPZ) and control groups. Control animals exhibited smaller CLs that remained from previous cycles, whereas treatment animals had larger CL per ovary


Corpora lutea (µm)	Control	3 mg/kg	10 mg/kg	30 mg/kg

301-400	0.75±0.25	0.20±0.00	0.25 ±0.25^a^	0.00 ±0.00^a^
401-500	2.00±0.00	0.75±0.25	0.25±0.25	0.25±0.25^a^
501-600	2.00±0.40	2.50±0.50	2.00±0.50	2.50±0.50
601-700	2.00±0.00	2.50±0.50	1.75±0.25	2.25±0.75
701-800	2.25±0.25	2.25±0.50	2.25±0.25	2.00±0.40
801-900	0.50±0.25	0.75±0.25	2.00±0.25^ a,b^	2.00±0.40 ^a,b,c^
900<	0.00±0.00	0.00±0.00	2.50±0.50	1.50±0.75^a,b^


^a, b, c^; Indicate significant differences (P<0.05) between CPZ groups with the control, 3 mg/kg and 10 mg/kg groups, respectively. All data are mean ± SD.

## Discussion

The present study attempted to reiterate and integrate the understanding of the well-known dose dependent adverse effects of a conventional antipsychotic agent (CPZ) on the reproductive system and functions in female rats mediated via the hypothalamic- pituitary-gonadal system.

Hormonal analyses showed increased serum prolactin and progesterone levels and decreased serum LH, FSH and E_2_ levels in rats that received CPZ. This observation was dose-dependent. On the other hand, histological and histomorphometric examinations showed that CPZ significantly enhanced atretic follicle formation which was accompanied by a remarkable decrease in the rate of normal follicles and significantly larger sizes of normal CL at the two high doses. The results of this study have demonstrated decreased potential fertility at the high doses of CPZ.

It is well established that dopamine plays a crucial role in tonic inhibition of prolactin secretion (18, 19). Dopamine acts on lactotroph cells in the anterior pituitary gland and inhibits prolactin secretion (20). In one study, the use of a dopamine antagonist, haloperidol, as an antipsychotic drug to inhibit dopamine secretion, has resulted in increased prolactin levels in rats (19). The results from biochemical analyses in our study corroborated with the mentioned hypothesis. Serum prolactin levels significantly increased in groups that received CPZ. This observation was dosedependent.

It has been reported that high prolactin levels inhibit the secretion of GnRH from the hypothalamus axis (21, 22). Prolactin can prevent luteolysis and cause increased numbers of persisting CL (23). The pulsatile secretion pattern of GnRH induces the cyclic release of LH and FSH. In female mammals, FSH induces follicle growth and subsequently E_2_ secretion by granulosa cells (24, 25). It has been reported that inhibition of GnRH results in reduced LH and FSH levels (26). Histological observations demonstrated that CPZadministered animals had significantly increased atresia of different sizes; these ovaries exhibited higher CL sizes. On the other hand, depending on dose, the serum level of E_2_ decreased and the progesterone concentration increased in CPZ-administered groups. Thus, it could be proven that increased levels of prolactin with a simultaneous effect of progesterone resulted in a remarkable follicular atresia. These impairments might not only be caused by higher prolactin levels, they might be caused with resistance CL from previous cycles (which, in turn leads to severe follicular atresia). This resistant CLs did not let the estradiol secretion restart, and reduced serum level of E_2_ in CPZadministered animals proofed mentioned theory very well. It is known that E_2_ directly stimulates prolactin synthesis in lactotrophs and prolonged E_2_ administration is known to produce elevation of serum prolactin levels and induce hyperplasia of prolactin-secreting cells. Even with the low levels of E_2_ that we have observed in the study rats that was attributed to hypogonadism, there was marked increase in prolactin secretion with CPZ treatment which showed the drug’s effect on prolactin secretion (27).

As previously mentioned, the increased level of prolactin can largely affect gonadotropins. Our analyses have shown that serum levels of LH and FSH significantly decreased in the two high CPZ dose groups. In patients treated with antipsychoticdrugs reduced secretion of GnRH in the hypothalamus decreased stimulation for LH and FSH secretion in the pituitary gland (11). Thus, we could conclude that CPZ directly and indirectly with hyperprolactinemia blocked the hypothalamus-pituitary axis, which in turn inhibited gonadotropin secretion. Additionally, the E_2_ positive feedback in the pituitary gland for LH hormone secretion was eliminated. Therefore the serum levels of LH and FSH decreased significantly in animals that received CPZ. Additionally, CL resistance delivered from the previous cycle caused decreased E_2_ level that was related with reduced gonadotropins and ultimately occurred situation increased atresia in CPZ-administered animals. Inhibited follicular growth marked with reduced normal follicles in CPZ-induced groups proved this theory.

In order to evaluate the biological activity of CLs, we investigated the serum level of progesterone. Observations demonstrated that the serum level of progesterone remarkably increased in animals treated with CPZ. This finding showed that the observed CLs were considerably active. Due to increased progesterone levels and absence of appropriate feedback for androgens and E_2_ secretion, in order to restart a new cycle (28, 29), follicular growth depression occurred in the ovaries of CPZadministered animals. A study suggested that estradiol actions on the oocyte or pregranulosa cells associated with the primordial follicle inhibited the initial wave of primordial to primary follicle transition. This decrease in primordial follicles in treated animals might be related in decreased E_2_ levels in these animals (30).

During the estrous cycle, E_2_ levels increase at proestrus and are low during estrus, metestrus and diestrus. Therefore in this study, we have observed that lower serum E_2_ levels in the treatment animals were consistent with the persistence of the diestrus phase (31). Hyperprolactinemia is known to be one of the causes of pseudopregnancy, namely continuous diestrus, by stimulating and maintaining CL in rodents since prolactin has a luteotropic activity (24). Thus, evidences can explain the reproductive disorders that have been observed in this investigation.

The luteotropic effect of prolactin, increase in progesterone and ovarian hormones, directly influence changes in the uterine wall. Remarkable (P<0.05) elevations have been observed in uterine horn endometrium, myometrium and perimetrium thicknesses along with remarkably higher gland number per mm^2^ of the endometrium in animals that received CPZ, which will be reported in another paper.

## Conclusion

Our results showed that rats treated with CPZ had mean serum prolactin levels several-fold greater than the upper limit of normal. Additionally, CPZinduced hyperprolactinemia was associated with a disturbance in the levels of essential reproductive hormones, E_2_ and progesterone. The prolactin-associated disturbances in gonadotropins and reproductive hormones exerted significant adverse effects on follicular growth in CPZ-administered rats. Accordingly, due to increased atresia at different follicle sizes (preantral and/or antral) in CPZ-treated rats and the absence of >500 μm follicles and increased CL size in the ovaries, it seemed that CPZ caused significant hypo-ovulation by increasing atresia. CPZ, as a prolactin-elevating antipsychotic drug, decreased the fertilizing index. This finding was particularly observed at higher doses. The mentioned impairments remarkably depended on CPZ doses.
